# Encouraging local ownership of an externally-coordinated capacity building initiative in Malawi, Mali, Mozambique, and Tanzania: an exercise in process evaluation

**DOI:** 10.7189/jogh.08.010304

**Published:** 2018-06

**Authors:** Emilia Vignola, Marcie Parkhurst, Amos Misomali, Rebecca Heidkamp

**Affiliations:** 1 Department of International Health, Johns Hopkins University Bloomberg School of Public Health, Baltimore, Maryland, USA; 2 FSG, Washington, D.C., USA; 3 Institute for International Programs, Johns Hopkins University, Lilongwe, Malawi

The Sustainable Development Goals echo the Paris Declaration on Aid Effectiveness, Accra Agenda for Action, and Millennium Development Goals (MDGs) in highlighting the need to build national capacity for monitoring and evaluation (M&E) as part of health systems strengthening [1-3]. An underlying principle of these frameworks is country ownership, the state in which local stakeholders exercise leadership over development policies, strategies, and actions. However, there is limited practical guidance available for implementing “country-owned capacity building”, particularly as many low- and middle-income countries continue to rely on external financial and technical support to implement these initiatives [4]. What are specific strategies external partners can use to encourage local ownership of M&E capacity building efforts, given the reality that most of these efforts are still not truly “country-owned”?

Process evaluation can help answer this question. While outcome evaluation determines whether a program was effective, process evaluation determines how and why a program is effective, by documenting program inputs and activities and assessing whether they meet program objectives [5]. Process evaluation is particularly helpful in assessing projects that consist of multiple interventions or that are being implemented across multiple settings, and should be conducted throughout project rollout as it allows implementers to identify obstacles and adjust activities before the project ends [6,7].

This viewpoint summarizes a midterm process evaluation of strategies designed to encourage local ownership of an externally-coordinated M&E capacity building initiative, the National Evaluation Platform (NEP), in Mali, Malawi, Mozambique and Tanzania. This article is part of a Journal of Global Health collection on NEP methods, results, and lessons learned.

## THE NATIONAL EVALUATION PLATFORM

The NEP is a systematic approach designed to strengthen public sector capacity in program evaluation related to maternal, newborn, and child health and nutrition (MNCH&N) [8]. The NEP is being implemented by the Institute for International Programs at the Johns Hopkins University (IIP-JHU) in partnership with government institutions in Malawi, Mali, Mozambique, and Tanzania. Funding for an initial four years (2013-2017) was provided by Global Affairs Canada. The four countries were chosen based on government interest in M&E capacity building and availability of institutional partners to host the program locally. They were also chosen to reflect geographic, linguistic, and epidemiologic diversity, to increase generalizability of project tools and lessons.

**Figure 1:**
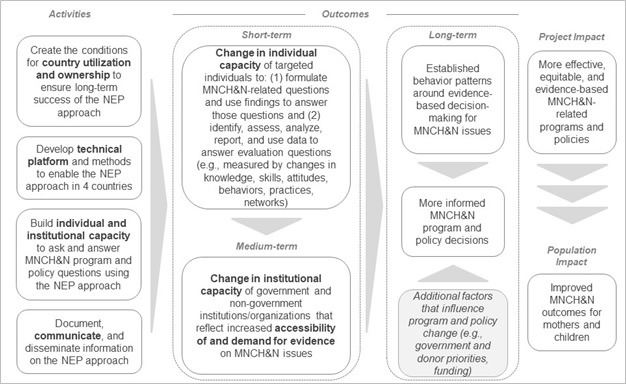
Original National Evaluation Platform Theory of Change.

The original NEP Theory of Change (TOC) (**Figure 1**) identified four areas of activities needed to meet the ultimate objective of strengthening evidence-based MNCH&N programs and policies:

- Create conditions for country utilization and ownership of the NEP approach;
- Develop the platform’s technical tools and methods;
- Build individual and institutional capacity to conduct MNCH&N research; and
- Document and communicate information on the NEP.

Specific strategies were then identified for each area of activities. Strategies intended to encourage ownership of the platform in each setting included (1) generating support for NEP among senior stakeholders and partner institutions; (2) engaging senior decision makers in the NEP approach; (3) establishing local structures and recruiting government staff; and (4) implementing NEP around the information needs of government stakeholders.

To support both process and outcome evaluations of the four-year project, IIP-JHU developed an internal documentation strategy that included event reports, staff interviews, and structured team reflections. Additionally, IIP-JHU commissioned the non-profit consulting firm FSG to conduct a midterm evaluation. Between May and September 2015, FSG conducted interviews and focus groups with JHU-IIP, as well as interviews, focus groups, and surveys with key project stakeholders in each NEP country. Below we reflect on successes and challenges in encouraging local ownership of the NEP identified through both internal documentation and the midterm evaluation.

## MIDTERM PROCESS EVALUATION: HOW WERE PROJECT STRATEGIES HELPING OR HINDERING THE DEVELOPMENT OF LOCAL OWNERSHIP?

### Generating support for NEP among senior stakeholders and partner institutions

In the project planning phase, IIP-JHU identified key in-country partners to engage given the governance structures they represented and given their personal interest in program evaluation. Senior IIP-JHU staff met personally with senior in-country stakeholders – including leaders of the Malawi National Statistics Office; the Malian Center for Research, Studies and Documentation for Child Survival; the Mozambican National Institute of Health; and the Tanzanian National Bureau of Statistics – to assess their interest in the initiative, discuss the project vision, and identify evaluation priorities. These discussions confirmed a desire for capacity building in evaluation and allowed IIP-JHU to identify early champions of the NEP. They also allowed in-country partners to participate in establishing NEP structures locally by determining whether specific committees were needed and advising on their membership. However, individual-level support did not translate automatically into institutional support, and it was not always sustained over time. Maintaining the support of early champions required continued effort, eg, seeking their strategic input throughout project implementation.

### Engaging senior decision makers in the NEP approach

After the planning phase, IIP-JHU sought to formalize senior stakeholder engagement in NEP by establishing a *High-Level Advisory* or *Steering Committee* (SC) in each country. The SC is led by government leaders, with participation by representatives of MNCH&N programs and institutions responsible for statistics, planning, and research. Beyond general oversight of NEP implementation, the SC is responsible for identifying priority evaluation questions relevant to the country’s MNCH&N needs; receiving and providing feedback on NEP analyses pertaining to those priority questions; and applying NEP findings to inform decision making. By the midterm evaluation, SCs in all four countries had met at least twice annually, bringing together stakeholders from a range of ministries that did not typically work together around important MNCH&N issues. However, the degree to which SCs had articulated clear evaluation priorities or decisions based on NEP findings varied across the four countries. One barrier was the logistical complexity of convening high-level stakeholders, which limited the frequency of SC meetings, making it difficult to keep members engaged over long periods. Local NEP teams adapted to this challenge by sending out routine project updates and in some cases by creating sub-groups of SC members who could meet more frequently to facilitate decision making of the broader committee.

### Establishing local structures and recruiting government staff

Other components of the NEP structure were designed so that local public-sector institutions played a central role in project implementation. Each NEP has a *Home Institution (HI)* – a national statistics office or public health research institute – that is responsible for day-to-day project management. HIs receive funding for staff and project activities, including workshops and dissemination events. Having respected governmental institutions take on this leadership role lent credibility to the project and helped to frame the NEP as a national resource. However, by the midterm evaluation, some of the challenges often associated with partnering with government institutions had become apparent. Most of the HIs had limited personnel and frequent turnover, with existing staff stretched across multiple projects. Contributing salary support for HI staff mitigated the challenge of limited human resources to some extent, as did communicating regularly with HI directors about the importance of protecting project staff’s time.

To facilitate collaboration between the US-based team and the HIs, IIP-JHU recruited *Resident Advisors* (RAs) who were nationals of each country. RAs are members of the IIP-JHU team who work closely with HI staff (three of the four RAs work from HI offices). As mid-career professionals actively engaged in the MNCH&N sector in their respective countries, all four RAs had an advanced understanding of local stakeholders’ interests, which helped external project staff navigate local politics. However, the degree to which each RA was perceived as being affiliated with the HI rather than IIP-JHU varied across the countries, and this occasionally created tensions for the RAs. The RAs also had limited power to impact bureaucratic processes, as they were outside government. The midterm evaluation highlighted the importance of being realistic about the power RAs wielded and of making sure the US-based team provided them adequate support.

IIP-JHU also worked with local partners to establish a *Technical Task Team* (TTT) at the center of each NEP structure, composed of staff from the HIs and the M&E units of stakeholder institutions. These groups are the target participants of NEP capacity building activities. Through workshops, small-group working sessions, and intensive mentoring, TTT members complete multiple “cycles” of data mapping and quality assessment, statistical analysis, and communication of results to respond to evaluation questions approved by the SCs. Though capacity building constituted a distinct set of activities within the NEP TOC, this aspect of the project facilitated local project ownership in several ways. Allocating resources to strengthening capacity of local staff signaled a commitment from IIP-JHU to support in-country partners in making their own evidence-based programming and planning decisions. Furthermore, TTT members developed effective collaborative relationships with each other; over time, most country teams worked more autonomously of IIP-JHU to complete analyses. Relationships built between TTT members also facilitated access to data and areas of expertise outside those of IIP-JHU, further enriching NEP as a national M&E platform. Despite these successes, consistency in participation throughout the learning cycles varied across the four countries, in some cases limiting the effectiveness of capacity building efforts. Planning activities more in advance and with greater frequency, eg, through working sessions between formal workshops, helped increase TTT member participation, especially when the sessions were coordinated autonomously by local NEP team members.

**Figure:**
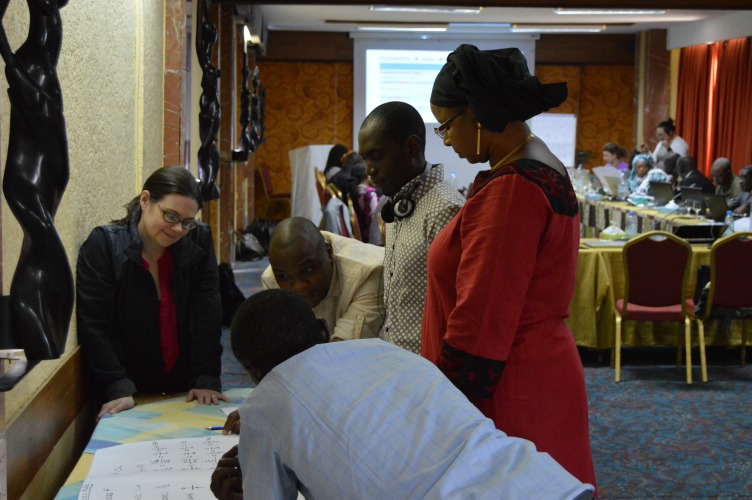
Photo: Project implementers and members of the Mali Technical Task Team at an NEP workshop (from the NEP collection, used with permission)

### Implementing NEP around the information needs of government stakeholders

Early in project implementation, senior stakeholders had difficulty grasping NEP’s value in concrete terms and did not initially express a demand for analyses. Therefore, for the first cycle, IIP-JHU proposed standard evaluation questions that would be useful to policymakers in the short term. NEP implementation began as MDGs were ending and as new planning cycles were beginning, so IIP-JHU proposed prospective program planning questions related to meeting national targets or retrospective questions about progress towards MDGs. After obtaining approval from each SC, all country teams analyzed coverage, mortality, and nutrition data from household surveys using the Lives Saved Tool to answer these questions [9]. Once SC members were presented with specific results, they understood more clearly what kinds of products could be generated through NEP. Results were used by government stakeholders in most settings, eg, to prioritize interventions in national health strategies, and dissemination of results was followed by requests for additional analyses. However, after this “quick win,” a tension emerged between satisfying government stakeholders’ needs and fulfilling project deliverables related to developing the NEP. For subsequent analyses, a less standardized approach was used across the four countries. In some cases, this allowed NEP analyses to be more responsive to stakeholder interests (as NEP is intended to function), but it also made it difficult to incorporate certain technical tools and analytical methods that IIP-JHU had committed to testing into country work.

## THE LONG ROAD TO TRUE COUNTRY OWNERSHIP

Process evaluation has been an important exercise for NEP implementers for several reasons. First, it helped NEP implementers develop a TOC that incorporated strategies to foster local ownership into the project from the start. Then, through systematic and ongoing assessment of project activities, it provided project implementers the opportunity to identify and respond to challenges along the way. Finally, the midterm evaluation serves as a benchmark to use in measuring progress by the end of the project. A forthcoming final evaluation will assess the degree to which NEP met its objectives, including that of fostering local ownership of the NEP approach. Additional areas to be assessed include capacity building outcomes, utility of technical tools, influence on decision making, and project sustainability.

We note two caveats. First, it is unrealistic to expect local ownership of an externally-led project to develop over four years. For that reason, the final evaluation will update the midterm evaluation with regards to progress towards fostering ownership, rather than make a definitive claim on whether ownership exists. Second, and more importantly, the NEP takes a step forward in channeling donor and foreign technical support in a way that encourages local ownership of efforts to improve MNCH&N. However, truly country-owned improvements in maternal and child health will require that in-country stakeholders have control over the design, implementation, and evaluation of projects, programs, and policies, as it is those in-country stakeholders who understand local needs and priorities best.
